# Bringing to Light Hidden Elasticity in the Liquid State Using *In-Situ* Pretransitional Liquid Crystal Swarms

**DOI:** 10.1371/journal.pone.0147914

**Published:** 2016-02-04

**Authors:** Philipp Kahl, Patrick Baroni, Laurence Noirez

**Affiliations:** Laboratoire Léon Brillouin (CEA-CNRS), CE-Saclay, 91191 Gif-sur-Yvette, France; University of California San Diego, UNITED STATES

## Abstract

The present work reveals that at the sub-millimeter length-scale, molecules in the liquid state are not dynamically free but elastically correlated. It is possible to “visualize” these hidden elastic correlations by using the birefringent properties of pretransitional swarms persistent in liquids presenting a weak first order transition. The strategy consists in observing the optical response of the isotropic phase of mesogenic fluids to a weak (low energy) mechanical excitation. We show that a synchronized optical response is observable at frequencies as low as 0.01Hz and at temperatures far away from any phase transition (up to at least 15°C above the transition). The observation of a synchronized optical signal at very low frequencies points out a collective response and supports the existence of long-range elastic (solid-like) correlations existing at the sub-millimeter length-scale in agreement to weak solid-like responses already identified in various liquids including liquid water. This concept of elastically linked molecules differs deeply with the academic view of molecules moving freely in the liquid state and has profound consequences on the mechanisms governing collective effects as glass formation, gelation and transport, or synchronized processes in physiological media.

## Introduction

Liquids differ from solids by a delayed response to a shear mechanical solicitation; i.e. they have no shear elasticity and exhibit a flow behavior at low frequency (< 1Hz). This postulate might be not verified at all length scale. Several recent experimental observations [1–8] highlight the existence of low frequency shear elasticity at the sub-millimeter scale supporting the hypothesis that the liquid state forms a resilient weakly bounded self-assembly. Consequently the flow behavior of liquids might be the product of the shear-melting of their primarily solid-like nature. The present work brings the robust evidence of the existence of shear-elasticity in liquids. The strategy is to “visualize” the solid-like dynamic response upon a low frequency mechanical excitation. For this, we use the birefringent properties of micron-sized precursors that some liquids contain at the approach of a first order phase transition. The isotropic phase of liquid crystals belongs to this category of fluids. Liquid crystals are formed of anisotropic molecules that are randomly oriented in the isotropic phase. Close to the isotropic transition, orientationnal pretransitional fluctuations coexist with the isotropic liquid. These precursors of the transition are swarms of locally ordered liquid crystal molecules [9]. The swarms are randomly distributed in the isotropic medium. On the assumption that the molecules (including the swarms) are elastically correlated in the liquid, the mechanical strain field should act on elastic correlations and orient the birefringent swarms that play the role of in-situ optical probe.

**Fig 1:**
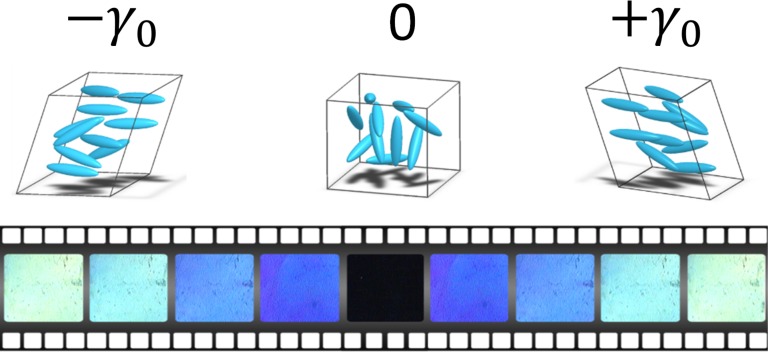
A low frequency mechanical excitation is applied to the isotropic phase of a liquid crystal. The photographs show that the liquid under stretching reversibly transits from a black to a bright state, revealing an unexpected elastic behavior. The snaphsots are recorded between crossed polarizers with a polychromatic light for one period of 0.5Hz and γ_0_ = 10%. The isotropic phase appears black at rest; γ_0_ = 0.

The article is constructed as followed. We first examine the optical behavior of the isotropic liquid of two liquid crystalline fluids upon low frequency mechanical strain. We reveal that it is possible to strain-induce a spectacular low frequency birefringence. This so far unknown property indicates that the isotropic liquid loses entropy to gain orientation (Fig 1). The analysis of the dynamic response indicates that the optical signal is synchronized with the stimulus and is stable up to 15°C above the transition over a wide frequency range (from 0.01 to several Hertz). The optical birefringent response reproduces a similar dynamic behaviour versus strain as previous stress measurements [1,3–8] and supports fully the assumption that liquids possess a shear elastic regime at low strain values prior to the conventional liquid or flow behavior.

## Materials & Methods

Two liquid crystalline fluids (LCF1 and LCF2) selected to represent the liquid behavior of a small molecule and of a large molecule, are examined in their isotropic liquid phase. LCF1 [3] is a 40 repetitive unit cyanobiphenyl ended side-chain polyacrylate of 4.4nm hydrodynamic radius and LCF2 [10] is a 12 repetitive unit cyanobiphenyl ended side-chain polyacrylate of 1.4nm hydrodynamic radius. They differ by a butyl (LCF1) and a propyl (LCF2) group presenting nematic to isotropic (LCF1) and smectic to isotropic phase transitions (LCF2) respectively. The isotropic phase of liquid crystals is usually devoid of interest because it presents the conventional properties of a Newtonian liquid (here LCF2) or of a viscoelastic melt (here LCF1) [9,11]. The symmetry and the thermodynamic characteristics (therein concerned isotropic-nematic and isotropic-smectic transitions are first order [9,12] and do not allow the miscibility of the ordered phase with the isotropic phase) are also those of a conventional liquid. Additionally, isotropization temperatures away from the glass transition or from the crystallization temperature (at about 60°C and 25°C for LCF1 and LCF2 respectively) prevent the isotropic phase from vicinity effects of the glass transition.

The optical experiments were performed in transmission mode under crossed polarized microscopy (Olympus BX60). Oscillatory shear motion was applied by a home-improved CSS450 Linkam cell (with a temperature gradient < +/- 0.05°C) equipped with quartz plate-plate fixtures. The used wavelengths were λ = 420nm and 614nm respectively. The transmitted intensity I was measured with a CCD camera (Vision Technology) and normalized to the uncrossed polarizer intensity I0. The averaged birefringence 〈Δn〉 is calculated using the formula $\frac{\text{I}}{{\text{I}}_{0}}={\text{sin}}^{2}(\langle \Delta \text{n}\rangle \frac{\text{e}\pi }{\lambda })$ where e is the gap thickness. A trigger device (by R&D Vision) measures simultaneously the movement of the lower plate and the transmitted intensity with two individual cameras (ALLIED Vision Technology). The trigger synchronizes very accurately the recording of the position and the movement and the transmitted intensity with a synchronization delay (Δτ = 10^-5^s). The strain amplitude is defined by the displacement length δl divided by the sample thickness(γ_0_ = δl/e). Typical displacements and strain amplitudes are of the order of 4 to 250μm for e = 250μm and γ_0_ varying from 2 to 100% respectively.

The stress measurements were carried out with a conventional device (ARES2) and via the simultaneous recording of the imposed strain signal (input wave) and of the sensor signal (output wave) with two multimeters plugged at each corresponding motor coupled to two 7-digits voltmeters (Keitley—Rate: 300data/s). This setup enables the simultaneous access to the strain/stress signals and to the dynamic profile versus frequency (ω) and versus strain amplitude (γ_0_). An optimized transfer of the motion to the liquid was achieved by ensuring total wetting boundary conditions between the liquid and the substrate (alumina plates of 20mm diameter) and by probing sub-millimeter gaps [1–8].

## Results

Based on the identification of a low frequency elastic regime that has been recently identified by stress measurements in different liquids [1–8] including the isotropic phase of liquid crystals [2,4], we examine the optical response of the isotropic liquid using similar strain and low frequency conditions. We suppose that applying a shear-strain induces a distortion of the elastic network. Similarly as optical trackers, the pretransitional swarms should reorient along the stretching direction “visualizing” the strain field. The aim is to access (reversible) elastic properties (the liquid is solicited below its flow threshold as for stress measurements). These linear nearly equilibrium conditions are quite different from strongly non-linear conditions producing the well-known flow birefringence of liquid crystals, micellar solutions [13–16] or even simple liquids [17]. These latest are theoretically described by irreversible thermodynamics [18, 19].

**Fig 2:**
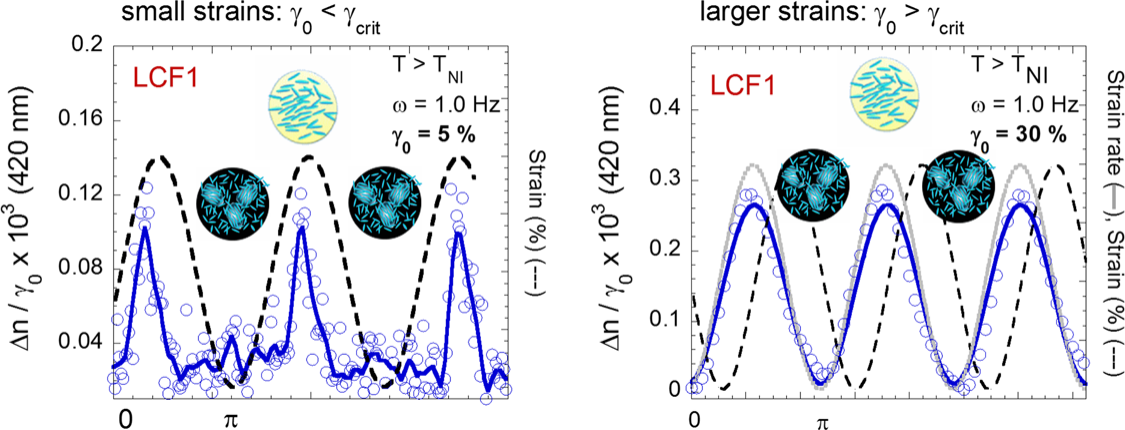
A mechanical oscillatory excitation at low frequency (ω = 1.0Hz) orients the pretransitional fluctuations in the isotropic phase (T-T_NI_ = + 0.5°C) and creates a reversible and defect-free birefringent signal (sample: LCF1, blue circles ○: sine model fit/smoothing of data points: continuous line, ▬). The birefringence values are normalized by the applied strain amplitude. **(a)** At small strain amplitude (γ_0_ = 5%), the optical signal is in-phase with the strain (black dashed line, - - -). (**b**) At larger strain amplitudes (here γ_0_ = 70%), the optical signal is synchronized with the strain-rate (continuous grey line, ▬).

**Fig 3:**
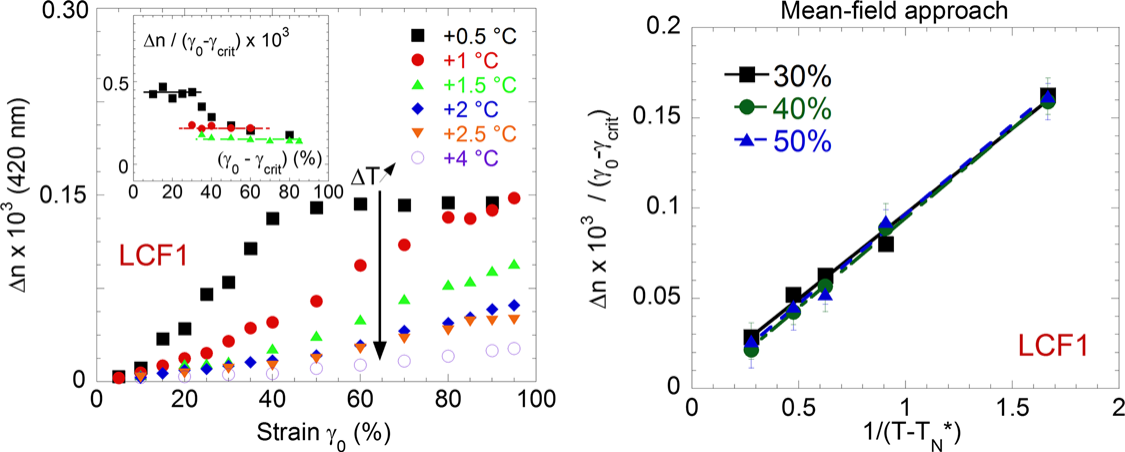
(a) Evolution of the low frequency birefringence (peak values) in the liquid phase versus strain amplitude γ_0_ at various temperatures. Sample: LCF1 thickness: 250 μm, ω = 1.0 Hz, at T-T_NI_ = 0.5°C (black squares,■), 1°C (red dots,●), 1.5°C (green triangles,▲), 2°C (blue diamonds,♦), 2.5°C (orange inversed triangles,▼) and 4°C (purple circle, ○). Insert: Reduced dynamic birefringence Δn_norm_ = Δn/(γ_0_-γ_crit_) versus strain amplitude. The eye-guide lines present a strain independent regime. (b) Mean field behavior of the low frequency birefringence at the approach of the transition temperature. The reduced dynamic birefringence is shown for three strain amplitudes γ_0:_ 30% (black squares,■), 40% (green dots,●) 50% (blue triangles,▲) as a function of 1/(T-T_NI_*) with T_NI_* = T_NI_ +0.4°C.

Fig 2 illustrates quantitatively the optical response of the isotropic phase of LCF1 to a low frequency mechanical field at small and at larger strain amplitudes γ_0_. The input signal is a sinusoidal shear strain wave of frequency ω and of amplitude *γ*_*0*_: *γ*(*t*) = *γ*_0_*sin*(*ωt*). A defect free reversible low frequency birefringence is observed at strain amplitudes as weak as 5%. The evolution of the birefringent signal follows a harmonic function of the input *sine* strain wave. It can be modeled by a *sine* wave *Δn* (*t*) = *Δn*_*max*_*sin*(*ωt* + *φ*) by which the phase shift *φ* in respect to the imposed strain wave is deduced. At small strain amplitudes (Fig 2(left)) below the flow threshold (*γ*_*0*_ < *γ*_*crit*_ with *γ*_*crit*_ *≈5%)*, the induced birefringence is weak and synchronized to the applied strain. At larger strain amplitudes (*γ*_*0*_ > *γ*_*crit*_*)*, the optical signal strongly increases becoming synchronized with the strain rate *dγ*(*t*)/*dt* = *γ*_0_*sin*(*ωt* + *φ*) (Fig 2(right)). Quantitatively the birefringence increases linearly with the strain amplitude (Fig 3A measured at various temperatures) before saturating at high strains. The linear regime is observed for a wide range of strain amplitude (tested from 10 to 100%), at various frequencies (0.01 to several Hertz) and a wide range of temperatures (up to 4°C above the isotropic-nematic transition for LCF1 and up to 15°C above the isotropic-smectic transition in the case of LCF2). The closer to the transition temperature, the more intense is the birefringence in agreement with the growth of pretransitional orientational fluctuations. By defining the quantity renormalized by the strain value ${\Delta \text{n}}_{\text{norm}}=\frac{\Delta \text{n}}{\gamma_{0}-\gamma_{\text{crit}}}$ as a reduced dynamic birefringence, we highlight the strain regime where the dynamic birefringence is independent of the applied strain amplitude (inset of Fig 3A). Being independent of the external stimuli, this linear regime defines a characteristic of the material; i.e. an intrinsic birefringence. The stimuli being the strain, the strain-birefringence curve (Fig 3A) is analogous to the stress-strain curve observed in solid mechanics displaying a linear elastic region at low strain followed by a plastic region at higher strain. Versus temperature, the low frequency birefringence (Fig 3B) obeys at low strain a typical mean field behavior: Δn_norm_~1/(T-T_NI_^*^) where T_NI_^*^- T_NI_ = 0.4°C confirming that the measurements are carried out in nearly non-perturbative conditions. Close to the transition T_NI_, a saturation of the induced signal is observed above strain amplitudes larger than γ_0_ = 50% (Fig 3A). The orientation has reached a maximum and the further strain increase is totally dissipative.

**Fig 4:**
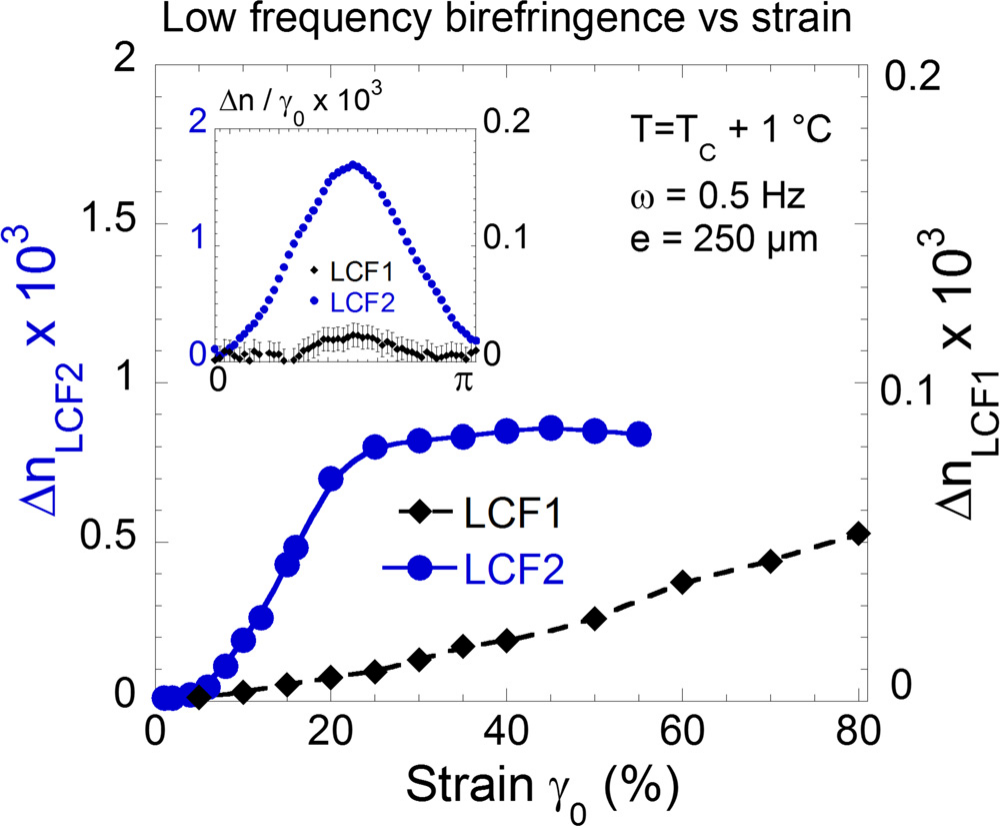
The small molecule LCF2 produces a stronger birefringence at low frequencies: Comparative intensities of the low frequency birefringence (peak values) displayed in the isotropic phase by LCF1 (black diamonds, ♦) and by the small molecule LCF2 (blue dots, ●) under the same conditions (ω = 0.5Hz, 250μm, +1°C above the isotropic transition) versus the strain amplitude γ_0_. The lines serve as eye-guides. Insert: Comparison of the optical wave signals of the isotropic phase of LCF1 and LCF2 at γ_0_ = 10% (double scale—the birefringence is normalized to the strain amplitude).

A similar behavior is obtained above the isotropic-smectic transition for the 12 repetitive unit liquid crystal (LCF2). Again, a splendid dynamic birefringence is observed in the isotropic phase. At low strain amplitudes (γ < γ_crit_), the signal is in phase with the strain while at larger strain amplitudes, the dynamic birefringence is in phase with the strain rate. The induced birefringence is measurable down to ω = 0.01Hz. At comparable frequencies the signal of LCF2 is about 100 times larger than for LCF1 (Fig 4 and insert). The difference might be explained by a stronger contribution of pretransitional fluctuations at the approach of the isotropic-smectic transition and by a transition to the isotropic phase about 45°C lower than for the nematic LCF1. The birefringence induced in the isotropic phase remains however weak about 1% of the value of commercial nematic displays [20]. Optimizing both chemical and physical parameters as the molecular structure or the anchoring may certainly improve these very first results.

**Fig 5:**
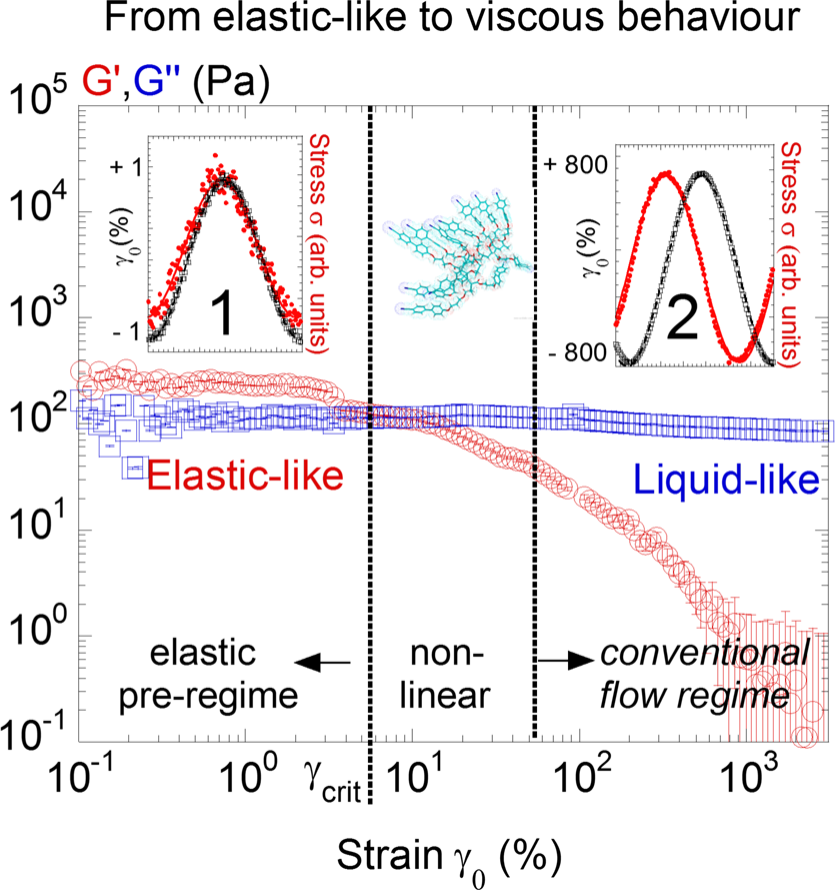
Identification of a elastic-like regime prior the Newtonian behavior in stress measurements: Stress dynamic moduli of LCF2 measured versus strain amplitude γ_0_ at +25°C above the isotropic-smectic transition and for a gap thickness of 170μm. Elastic modulus G’ (red circles, ○) and viscous modulus G” (blue squares, □) at a frequency of ω = 0.5rad s^-1^. The vertical dashed bar delimits the elastic regime (elastic-like behavior) at small strains γ_0_ from the flow regime (liquid-like behavior) at large strains γ_0_. Insets: Raw periodic stress (red full dot: •) and strain (black hollow circles: ○) signals at small and large strain amplitudes (γ_0_ = 1.0% and at γ_0_ = 800% respectively).

**Fig 6:**
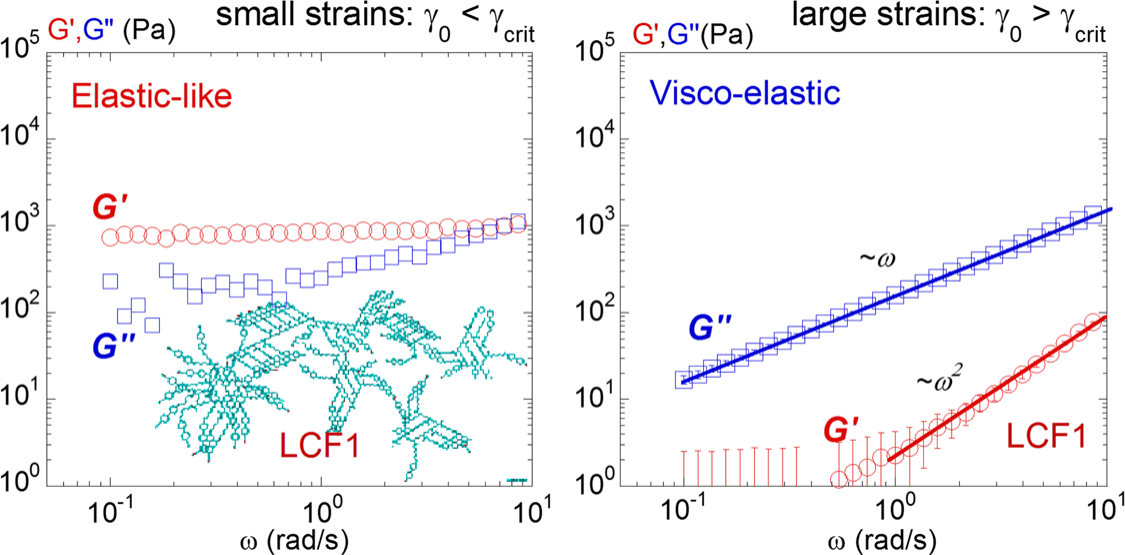
Elastic-like versus liquid-like relaxation spectra of LCF2: (left) At small strains (γ_0_ = 0.5%) the elastic modulus G’ (red circles, ○) and the viscous modulus G” (blue squares, □) are independent of the frequency. G’ is the dominating modulus indicating an elastic-like behaviour. (right) At large strains (γ_0_ = 300%) the viscous modulus shows a conventional behaviour (G” = ω.η) and reveals a liquid-like behaviour. Measurements carried out at +25°C above the isotropic-smectic transition, gap thickness:170μm.

We now compare the optical response in the frame of stress measurements. This stress response can be expressed in terms of frequency-dependent shear moduli where the elastic modulus G’(ω) and the viscous modulus G”(ω) are the Fourier transform of the stress relaxation function G(t) of the quiescent state: G’(ω) = ω$\int_{0}^{\infty }G\left(t\right)sin\omega t.dt$, G”(ω) = ω$\int_{0}^{\infty }G\left(t\right)cos\omega t.dt$. When the response is linear with respect to the strain solicitation, G’ and G” describe characteristic properties of the material. G’ > G” indicates a rather solid-like while G” > G’ indicates a viscous or flow behavior. Liquids and viscoelastic liquids are characterized by a flow behavior at low frequency, typically within 0.1 – 10^2^Hz. However recent improvements in dynamic analysis [1–8] have revealed that it is possible to access a finite shear elasticity in the low frequency domain not only close to a surface [21] but also at a sub-millimeter length scale in liquids as little viscous as liquid water [7] or isotropic phases of small liquid crystal molecules and in liquid crystal polymers including LCF1 [1–5]. Fig 5 and Fig 6 summarize the stress behavior of the isotropic phase of LCF2.

**Fig 7:**
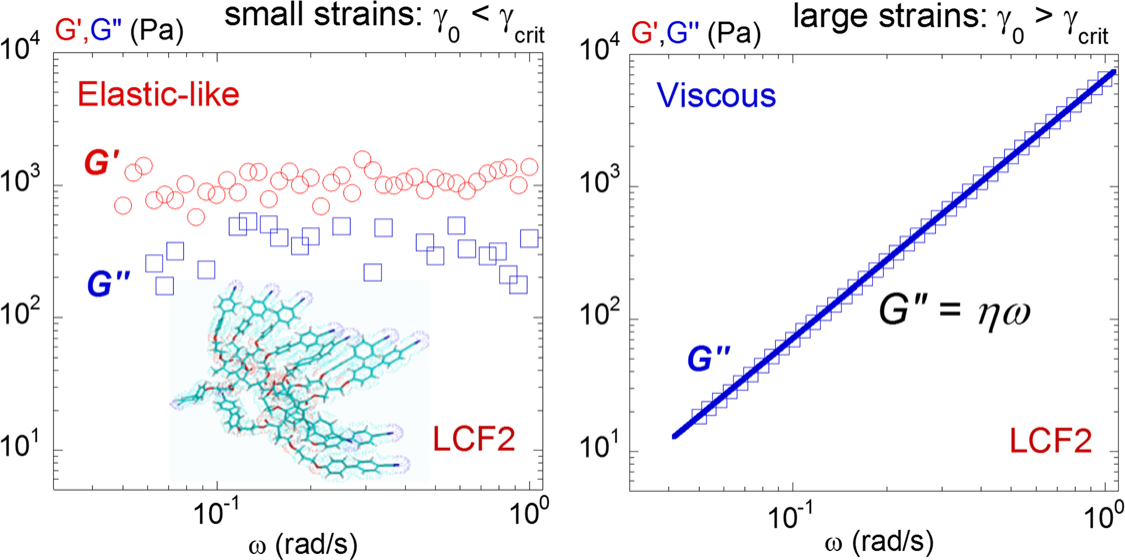
Elastic-like versus viscoelastic relaxation spectra of LCF1: (left) At small strains (γ_0_ = 1.0%) the dynamic response of the LCF1 is similar to LCF2. The elastic modulus G’ (red circles,○) dominates the viscous modulus G” (blue squares, □) both being independent of the frequency indicating an elastic-like behaviour [3]. (right) At large strains (γ_0_ = 100%) the viscous modulus shows a conventional behaviour (G” = ω.η) and reveals a viscoelastic behaviour. Measurements carried out at +6°C above the isotropic-nematic transition, a gap thickness: 200μm.

Fig 5 displays the evolution of the dynamic moduli as a function of the strain amplitude γ_0_ above the isotropic transition. Similarly as in previous studies [1–8], an elastic pre-regime is identified prior to the conventional flow regime. Below a critical threshold at small strain amplitude (γ_0_ < γ_crit_), the superposition of the stress/strain signals indicates an almost instant response (left upper insert (1) in Fig 5); the isotropic phase behaves solid-like. Upon increasing the strain amplitude, the stress/strain signals become progressively phase shifted. At very large strain amplitudes (γ_0_ > 300%), the phase shift reaches π/2 which corresponds to the definition of a flow behavior. In terms of viscoelastic moduli, at large strains, the elastic modulus G’ drops rapidly while G” is nearly constant leading to a strain-induced transition from a solid-like to an apparent liquid-like behavior; i.e. away from equilibrium conditions (in agreement with strain behaviors observed on other liquids [4–8]). Fig 6 (left and right) detail the frequency dependence at low and at high strain amplitudes respectively. At small strain amplitude (γ_0_ = 0.5%), *G’* and *G”* keep constant values with the elastic modulus G’ dominating the viscous modulus G” (Fig 6. (left)) confirming the solid-like character over more than a frequency decade. At large strain amplitude (γ_0_ = 300%), G” scales with ω while G’ is undetermined (Fig 6 (right)). The isotropic phase of LCF1 shows a comparable dynamic behavior. At low strain amplitude (γ_0_ = 1.0%), the elastic modulus G’ dominates the viscous modulus G” both being independent of the frequency (Fig 7 (left)). At high strain amplitudes, the Newtonian behavior observed for LCF2 is here replaced by a conventional viscoelastic flow regime (Fig 7 (right)[3].

## Discussions–Conclusions

A so far unknown birefringence is identified in the liquid state of a large and a small liquid crystals, visible up to 15°C above the isotropic transition, at low frequency (tested from 0.01Hz up to 2.0Hz), and over a wide strain range. This low frequency birefringence whose origin is coupled to the orientation of the orientational pretransitional swarms appears at much larger timescales than known lifetimes τ_N_ measured by Kerr effects in the isotropic phase. These are of the order of τ_N_ ≈ 10^−9^s for the orientational pretransitional fluctuations of rod-like liquid crystals [22], necessitating frequencies of about 10^9^Hz to induce a coupling with these time scale. The lifetimes measured by Kerr effect in high molecular weight liquid crystals are lying around τ_N_ ≈ 10^−4^s [23] which still demand MHz solicitation frequencies.

The present study explores a tenth of Hz range via a mechanical action. This spectacular optical effect is not explainable on the basis of the above pretransitional lifetimes. It is approached by assuming the existence of low frequency shear elasticity in the liquid state leading to an opto-mechano coupling. The shear elasticity is measurable at the submillimetre length-scale at low strain values prior to the known flow behavior (Fig 5) [1–8]. The dependence of the low frequency birefringence with the strain exhibits similarities with the strain-stress behavior: it is in-phase with the strain at very low strain values (the elastic regime) and in-phase with the strain rate at higher strain values (γ_0_ > ca. 5%) corresponding to the entrance in the flow regime. Similarly, the linear relationship between the strain and the birefringence curve (Fig 3A) characterizes typically an elastic behavior. But the birefringence indicates that a long range ordering is established. This ordering rules out the condition of an isotropic liquid. Therefore the Newtonian behavior of the stress curve does not correspond to a flow regime but to a long range oriented state and thus to an entropic loss. The energy induced during the strain is accumulated to produce an ordered phase (orientation of the pretransitional swarms). This is a delayed process that decreases the entropy and that releases once the strain is removed. The isotropic phase behaves as a weak elastic material alternating entropic loss and recovery during the backward-forward motion [24]. This mechanism transforms the accumulation of the energy in an ordering (entropy loss during the stretching phase) and its recovery to the initial state (entropy gain during the relaxing phase) explaining the synchronization with the shear strain rate. This result is possible if the pretransitional swarms are embedded within the elastic net and respond reversibly to the stress field playing the role of a dynamic optical probe.

The present results confirm that the isotropic phase is an elastically correlated “self-assembly” in agreement with stress measurements indicating a weak elastic modulus at low frequency [1–8]. In light of this, the flow regime can be considered as an entropy-driven transition from an isotropic elastic state to an (anisotropic) oriented state. The elastic correlations between molecules are not lost but directed. Only few theoretical developments set the role of intermolecular interactions as crucial [25–27]. Noteworthy approaches consider a solid-like continuum in molecular liquids [28] and predict the shear-elasticity at several molecular layers scale [29], macroscopically [27], or depending on the length scale [30]. The shear elasticity is usually not considered since this delicate signal mostly observable at low thickness geometry is hardly experimentally measurable and is hidden in conventional mechanical measurements. It is however not negligible. Here the consideration of the low frequency elasticity makes possible the identification of novel properties. The strong birefringent signal at low frequencies in the isotropic liquid phase is obviously an attractive property reversibly converting a mechanical action in an optical device from a true black state to a birefringent state.
